# Bioengineering strategies to control epithelial-to-mesenchymal transition for studies of cardiac development and disease

**DOI:** 10.1063/5.0033710

**Published:** 2021-04-23

**Authors:** Dawn Bannerman, Simon Pascual-Gil, Marie Floryan, Milica Radisic

**Affiliations:** 1 Department of Chemical Engineering and Applied Chemistry, University of Toronto, Toronto, Ontario M5S 3E5, Canada; 2 Institute of Biomedical Engineering, University of Toronto, Toronto, Ontario M5S 3G9, Canada; 3 Toronto General Hospital Research Institute, University Health Network, Toronto, Ontario M5G 2C4, Canada; 4 Department of Mechanical and Industrial Engineering, University of Toronto, Toronto, Ontario M5S 3G8, Canada

## Abstract

Epithelial-to-mesenchymal transition (EMT) is a process that occurs in a wide range of tissues and environments, in response to numerous factors and conditions, and plays a critical role in development, disease, and regeneration. The process involves epithelia transitioning into a mobile state and becoming mesenchymal cells. The investigation of EMT processes has been important for understanding developmental biology and disease progression, enabling the advancement of treatment approaches for a variety of disorders such as cancer and myocardial infarction. More recently, tissue engineering efforts have also recognized the importance of controlling the EMT process. In this review, we provide an overview of the EMT process and the signaling pathways and factors that control it, followed by a discussion of bioengineering strategies to control EMT. Important biological, biomaterial, biochemical, and physical factors and properties that have been utilized to control EMT are described, as well as the studies that have investigated the modulation of EMT in tissue engineering and regenerative approaches *in vivo*, with a specific focus on the heart. Novel tools that can be used to characterize and assess EMT are discussed and finally, we close with a perspective on new bioengineering methods that have the potential to transform our ability to control EMT, ultimately leading to new therapies.

## I. INTRODUCTION

Epithelial-to-mesenchymal transition (EMT) was first identified by Hay in the last decade of the 20th century.^1,2^ According to this initial description, the EMT process involves the transition of epithelial cells—organized in sheet-like arrangements of polarized cells connected with tight and adherent junctions—into mesenchymal cells. The adoption of the mesenchymal cell phenotype implies enhanced migratory capacity and plastic properties. Invasiveness, mobility, and the ability to differentiate into other cell types are the main characteristics of the newly formed mesenchymal cells.^3–5^ Although the definition of EMT has not changed over the years, EMT was initially described to be a fundamental one-directional process in development^1^ and fibrotic processes.^2^ Since then, new evidence has confirmed that EMT is reversible, and mesenchymal-to-epithelial transition (MET) is also possible and contributes to reprograming and the generation of different epithelial organizations.^6^ Moreover, current knowledge confirms the role of EMT not only in early development (i.e., during embryonic stem cell differentiation, mammalian implantation, gastrulation, and neural crest development)^4,7^ and tissue fibrosis,^8,9^ but also in a number of biological processes during a lifetime, including tissue repair,^10^ wound healing,^11^ stem cell behavior,^12^ and tumor progression.^13^ The main reason EMT is such a relevant biological process is that it is involved not only during embryogenesis but also during disease and fibrosis, representing a hot topic for researchers looking for disease treatment approaches, as demonstrated by over 5700 publications indexed by Web of Science in 2019 alone.^14^ Indeed, EMT is generally classified into three subtypes based on the processes it is involved in. Type 1 EMT is involved in implantation, embryogenesis, and organ development, type 2 EMT occurs during wound healing, tissue regeneration, and fibrosis, typically following injury, and type 3 EMT occurs in cancer progression and metastasis.^5,15^

In the heart, it is well known that EMT contributes to organ development, whereas its importance in the adult heart and disease has been under investigation more recently.^16^ In mammals, the heart is divided into three layers, from inside to outside: endocardium, myocardium, and epicardium. In the developing heart, cardiac progenitors undergo several waves of EMT/MET/EMT, and the resulting mesenchymal cells migrate toward the myocardium and transition into other cardiac cells such as fibroblast, smooth muscle, and according to some reports even a small number of endothelial cells,^17–19^ giving rise to the different cardiac layers. Then, EMT events contribute to valve formation and final heart septation.^20^ In this regard, the architectural rearrangements and cellular changes necessary for valve formation are further supported by endothelial to mesenchymal transition (EndMT) processes.^21^ After heart organogenesis, the epicardium has been shown to be the center in the signal exchange between the epicardium and the myocardium,^22^ controlling mesenchymal cells' fate through the secretion of specific growth factors such as transforming growth factor-β (TGF-β), fibroblast growth factor (FGF), and PDGF, among others. In addition, during development, paracrine signals derived from the epicardium stimulate the growth of the underlying myocardium.^23^ Thus, the epicardium is vital for normal development of the heart and coronary vasculature.^24–27^ Whereas MET is the opposite process of EMT, EndMT is very alike. In fact, there is clear evidence indicating EndMT is involved not only in cardiac development like EMT but also in adult cardiovascular diseases such as atherosclerosis, valvular disease, fibrosis, and fibroelastosis.^28,29^ As expected, both EMT and EndMT present several similarities, for instance, regulatory pathways relying on transforming growth factor-β (TGF-β) signaling.^30^ Nevertheless, EndMT gives rise to mesenchymal cells that mainly assist valve development or contribute to fibroblast populations in the adult heart,^31^ while EMT is capable of giving rise to a more diverse population of cells as reviewed below. Moreover, taking into account the relevance of the epicardium as a source for paracrine signaling^22^ and as a mediator with the immune system,^32,33^ we will prioritize heart EMT over EndMT or MET in this review.

After heart development is complete, EMT does not normally reactivate. Only under specific conditions involving severe injury, for instance, myocardial infarction (MI), EMT is partially induced again in the epicardium as a defensive mechanism to produce mesenchymal cells. These mesenchymal cells will then principally differentiate into fibroblasts, as demonstrated using lineage tracing (Tcf21:CreER, that labels epicardial cells and their progeny) in zebrafish models.^34–36^ It is therefore believed that EMT in response to MI mainly causes fibrosis and scar formation to avoid left ventricular wall rupture, with no substantiated evidence that epicardial EMT replenishes cardiomyocytes in the infarct zone, as reviewed here.^16,35,36^ Despite mice work confirming this trend toward fibroblasts differentiation,^16,22^ other experiments postulated that epicardial EMT is also involved in the formation of perivascular cells^20,36^ and cardiac progenitor cells,^37,38^ contributing to several cardiac lineages and supporting tissue repair after injury.^39^

Finally, emerging evidence strongly suggests that there is a crosstalk between the epicardium and the immune system. In particular, the epicardium is required for yolk sac macrophage engraftment in the developing heart.^32^ These macrophages will then constitute the population of tissue-resident macrophages in the adult heart and promote cardiac tissue homeostasis, blood vessel formation, cardiomyocyte proliferation, and cardiac tissue electrical properties.^40–42^ Recently, the epicardium has also been shown to mediate an immunosuppressive response to MI through paracrine signaling that increases regulatory T cell recruitment to the heart.^33^ As a matter of fact, the inflammatory process that takes place in the heart after MI plays a critical role in tissue repair and can foster infarct progression or induce cardiac healing and regeneration.^43,44^ Consequently, it is expected that EMT in the adult heart may have effects on the inflammatory process after MI through directly modifying the epicardium, thus affecting tissue reparative processes. Altogether, this highlights the potential of EMT as a therapeutic target for MI and confirms the need to further understand how to tune and control this biological process in order to obtain the desired beneficial effects without triggering undesired side effects.

In order to study cellular processes such as EMT, cells are cultured on tissue culture plates. However, it is now well established that two-dimensional (2D) cell culture systems do not completely recapitulate the *in vivo* systems that they are attempting to mimic, with simplicity and immature cell type being potential issues.^45^ The addition of extracellular matrix (ECM) components or scaffolds, numerous cell types, spatially and temporally controlled chemical or biological conditions or stimuli, tuned mechanical properties, and additional physical factors, are all important aspects to incorporate in order to mimic native tissue environments. 3D cell culture systems, often referred to as organ-on-a-chip platforms, are able to include some of these additional properties and generate a more wholistic view of the systems involved.^46,47^ When it comes to understanding a process such as EMT, tools to recapitulate it more accurately should be employed. As more is understood regarding the mechanisms and implications of EMT, investigations can include bioengineering concepts to a greater extent, with more focus on biomaterial design, mechanobiology, and spatiotemporally controlled delivery of factors. While EMT is already inherent in stem cell differentiation protocols, perhaps its involvement in generating more complex 3D tissue constructs will be useful going forward. If sufficiently controlled, the power of the EMT process could be used to generate desirable tissue for tissue engineering purposes and ideally, this process could be precisely controlled *in vivo* to facilitate tissue regeneration. Of particular interest *in vivo* is achieving enhanced control over the EMT process in the epicardium of adult tissue, as this could lead to novel routes for cardiac recovery following injury, for example, through the promotion of transition into cell types that support reparative processes (e.g., vessel formation) over pathological processes (e.g., fibrosis).

In this review, we will describe the EMT processes that are relevant from a clinical point of view, with a specific focus on the heart, and review bioengineering strategies that have been used for controlling and studying EMT. Finally, we will discuss new methods for measuring EMT and discuss future directions to enhance EMT control.

## II. CELLULAR PROCESSES INVOLVED IN EMT

**FIG. 1.**
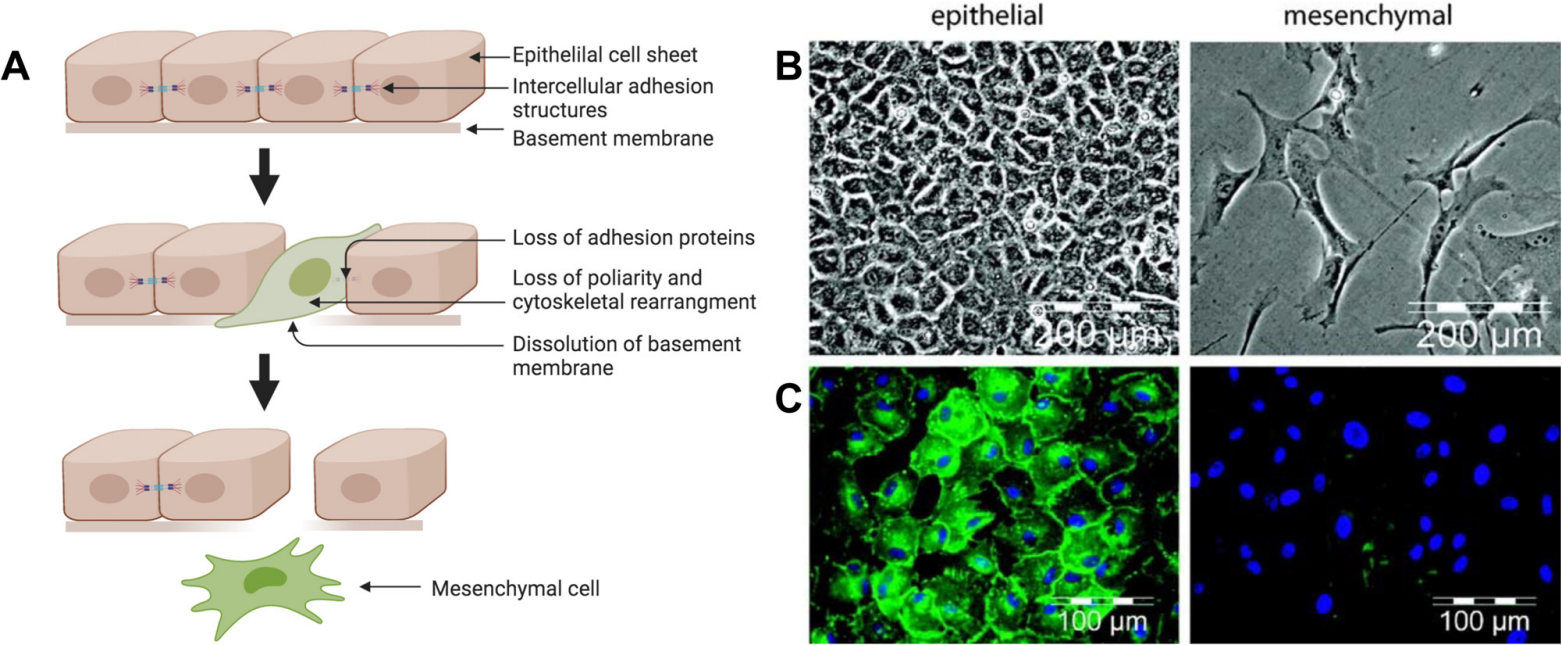
Cellular changes in EMT. (a) An epithelial cell sheet undergoes EMT. Loss of adhesion proteins, dissolution of the basement membrane, and changes in protein expression and cell structure all take place, facilitating the progression from epithelial cell type to mesenchymal cell type. Figure created with BioRender.com. (b) and (c) EMT in epicardium-derived cells showing epithelial morphology (left) and mesenchymal morphology (right). Images include bright-field (b) and immunofluorescence (c) with β-catenin stain shown in green and Hoechst 33342 stain of nuclei shown in blue.^58^ Reprinted with permission from van Tuyn *et al.*, Stem Cells **25**, 271 (2007). Copyright 2007 AlphaMed Press and John Wiley and Sons.

The transdifferentiation of polarized epithelial cells into motile mesenchymal cells facilitates the generation of new tissues during embryogenesis and organ development.^48,49^ It has also been reported in cancer development and metastasis as one of the earliest events in tumor progression.^50,51^ Cells undertaking EMT must undergo several changes that allow them to transform from the epithelial phenotype to the mesenchymal phenotype and become migratory, shown in Fig. 1. Upon initiation of EMT, repression of the transcription of adhesion proteins takes place and epithelial cell–cell junctions that are essential for epithelial integrity are deconstructed.^52^ Thus, the dissolution of intercellular adhesion junctions, specifically tight junctions (TJ), adherens junctions (AJ), desmosomes at lateral surfaces, and scattered gap junctions at lateral surfaces, occurs.^52,53^ The loss of E-cadherin, which is a calcium-dependent transmembrane glycoprotein that plays a main role in cell–cell adhesion and participates in intracellular signaling pathways, is a critical event in EMT. Notably, the presence of E-cadherin at the cell surface is distinct from that in the cytoplasm, and reorganization to the cytoplasm resulting from post-translational mechanisms has been associated with progression along EMT.^54,55^ Reversibility is a key feature of EMT and is a result of the ability for cell adhesion proteins to recycle in and out of junctions.^53^

Another main cellular change that occurs in EMT is the loss of apical-basal polarity, which occurs through signaling that disrupts polarity along with TJ disassembly, followed by transcriptional repression of polarity molecules.^53^ As cells lose their polarity, further rearrangements in the cytoskeleton occur. Downregulation of epithelial markers can be observed, and upregulation of mesenchymal proteins occurs.^56^ Additionally, extracellular matrix remodeling enzymes, matrix metalloproteases, are expressed which enables the breakdown of the basement membrane.^57^ Finally, cell invasion and migration can begin to take place. The cells resulting from EMT are motile and have increased cell contractility, cell protrusions, and actin stress fiber formation.^56^ Smooth muscle actin (SMA) expression is often a marker of the final stages of EMT.

## III. SIGNALING PATHWAYS AND FACTORS INVOLVED IN EMT

The molecular mechanisms and signaling pathways that control EMT in scenarios of both organ development and disease are the same, including the master transcription factors Snail, Slug, Twist, and zinc finger E-box-binding homeobox (ZEB) family.^59^ Their expression is activated early in EMT and controlled by well-known signaling pathways such as mitogen-activated protein kinase (MAPK)/extracellular signal-regulated kinase (ERK), signal transducer and activator of transcription (STAT), phosphatidylinositol-3-kinase (PI3K), and GLI.^60,61^ E-cadherin is the major component of epithelial junctions, and its repression is mainly mediated by SNAIL, SLUG, TWIST, and ZEB.^62^ It is important to note that other signaling pathways (i.e., Notch through the E47 protein) could also negatively regulate E-cadherin expression^63^ and contribute to EMT. Similarly, new progress is being made in the activators of the signaling pathways involved in EMT. For instance, there is a large body of evidence indicating that (TGF-β) (and associated Smad proteins) are the most potent inducers of the transcriptional repressors of E-cadherin.^64–67^ More recently, SETD1B was identified as a new ZEB1 target *in vitro* and *in vivo* with implications in EMT during colorectal cancer,^68^ confirming the complexity of the EMT process and the need to further explore EMT in order to fully understand the mechanisms involved. The downregulation of E-cadherin is paralleled to the upregulation of N-cadherin, the other major consequence of the transcriptional machinery triggered during EMT. The loss of epithelial E-cadherin and the gain of mesenchymal N-cadherin expression, known as the cadherin switch,^60^ is a major hallmark of EMT since the gain of N-cadherin expression provokes cytoskeletal changes that increase cell migration, motility and invasion.^52,69^

Scoping down, there is increasing evidence of the role that mechanical forces and interactions play in initiating these described EMT signaling pathways. For instance, it has been reported that an increasing matrix stiffness promotes EMT through the promotion of the nuclear translocation of TWIST1 from its cytoplasmic binding partner G3BP2.^70^ A study of oral squamous cell carcinoma (OSCC) showed that, after being exposed to a mechanically stiff niche for a prolonged time, a less invasive OSCC line characterized by a high E-cadherin to N-cadherin ratio increased migration speeds, suggesting that OSCC cells are mechanically sensitive and their tumor progression is in part mediated by this sensitivity.^71^ The relevance of mechanical forces as starting agents for EMT was also tested in different *in vivo* models, such as *Drosophila,* showing that an important driving force in EMT on epithelial morphogenesis occurs at the initiation of the EMT process in which cells produce an apico-basal force orthogonal to the surface of the epithelium.^72^ In fact, such apico-basal force is directly connected to the ECM – basal lamina.^48^ The ability of ECM to trigger the mechanotransduction was clearly demonstrated as when ECM laminin specific integrin receptors α3β1 and α6β4 are deleted, EMT activation was decreased by downregulation of EMT-linked focal adhesion kinase (FAK), Rac1, MAPK, and c-Jun NH2-terminal kinase pathways.^73^ In correlation to this, other studies of alveolar epithelium^74^ and hepatocellular carcinoma^75^ showed a synergistic effect between α3β1 and TGF-β1, resulting in an increase in EMT mainly due to a upregulation of SMAD, SNAIL, and SLUG pathways. Collagen IV, other ECM component, can also affect EMT by inducing epithelial repressors SNAIL and SLUG in a process mediated by FAK/ERK signaling and NFκB activation.^76^

**FIG. 2.**
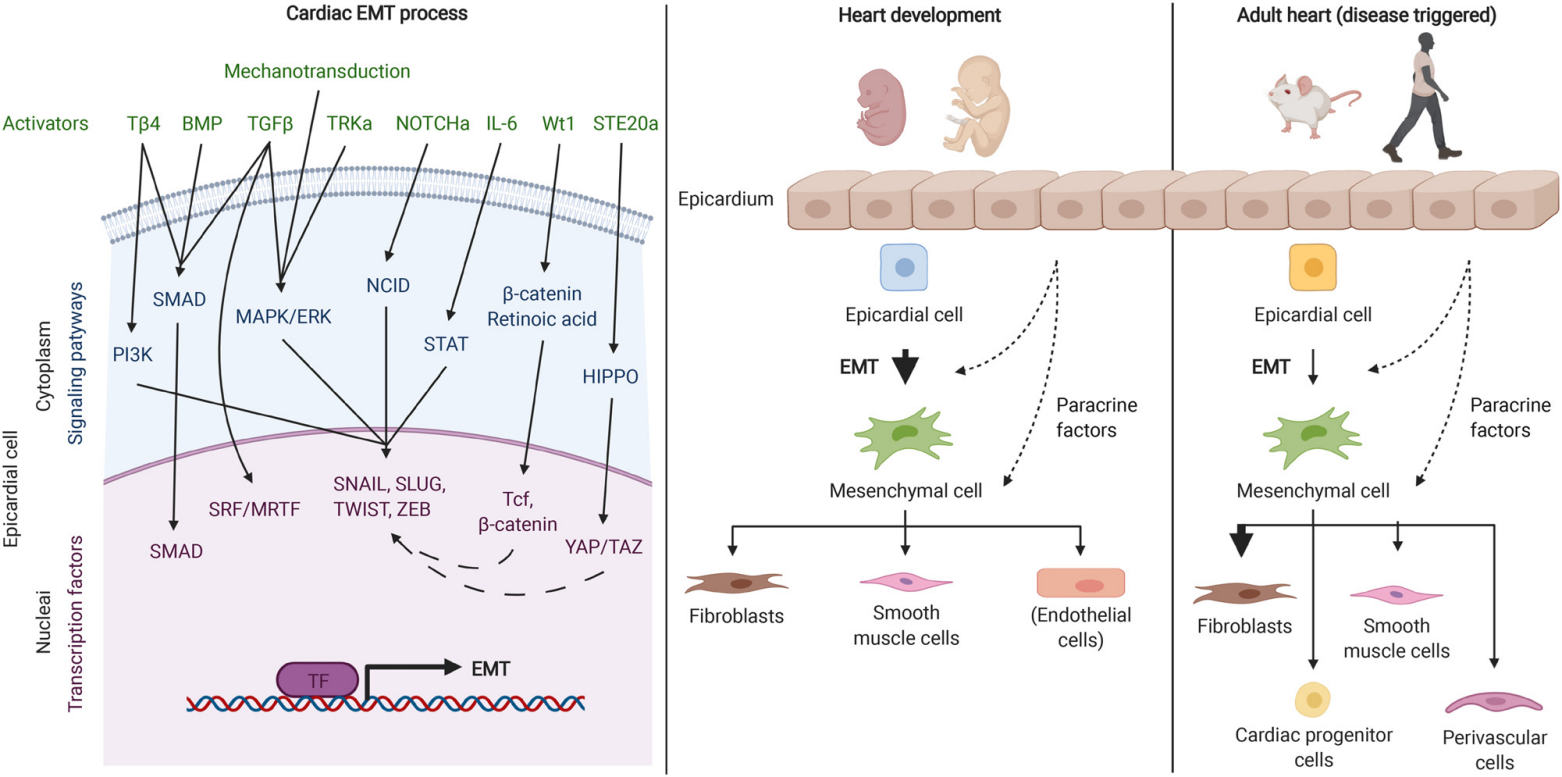
Endothelial-to-mesenchymal transition in the heart. The left panel represents the main activators (in green), signaling pathways (in blue) and transcription factors (in purple) that control the EMT process in the heart. The middle panel is a schematic of the EMT during development, and how it contributes to the generation of other cardiac cells types in the heart (brackets represent a minor population). The right panel shows cardiac EMT in adult hearts. In adults, EMT is triggered after cardiac diseases such as acute myocardial infarction. After suffering from myocardial infarction, epicardial cell undergo EMT. However, epicardial cells are in a different state compared to development (blue vs yellow colors). Although EMT is a normal process in the neonatal heart (indicated by thick arrow), it is not as efficient and common in the adult. Moreover, adult EMT mainly give rise to fibroblasts, whereas the other potential cell products are scarce. Tβ4: thymosin β4. BMP: bone morphogenetic proteins. TGFβ: tumor growth factor β. TRKa: tyrosine kinase receptor activators. NOTCHa: NOTCH signaling pathway activators. IL-6: interleukin 6. Wt1: Wilms' tumor suppressor gene1 protein. STE20a: sterile 20-like protein kinase activators. Figure created with BioRender.com.

In terms of the heart, during development, epicardial cells undergo EMT and constitute a source of multipotent progenitors for cardiac lineages^77,78^ (Fig. 2). Although traditional transcription factors (SNAIL, SLUG, and TWIST^16,79^) and growth factors (TGF-β^80^) are involved in epicardial EMT, in the cardiac environment, the transcriptional regulator Wilm's Tumor Gene 1 (Wt1) and the Hippo^81^ signaling mediators YAP and TAZ have also been reported to be the critical factors that control epicardium EMT (Fig. 2). Thus, Wt1 is essential during embryonic stem cell differentiation, directly inducing SNAIL, inhibiting E-cadherin expression and upregulating retinoic acid (RA), which is crucial for cardiac patterning and morphogenesis.^82,83^ Moreover, canonical Wnt/β-catenin signaling pathway is known to contribute to embryonic development,^84^ and physical or genetic ablation of Wt1 leads to defects in coronary vessel formation and impaired cardiomyocyte proliferation.^83^ On the other hand, inhibition of HIPPO signaling mediators YAP and TAZ during development lead to impaired epicardial EMT and reduced epicardial cell proliferation and differentiation into coronary endothelial cells.^85,86^ The regulation of the activity of the HIPPO effectors YAP/TAZ is controlled by modifications of the composition and mechanics of the ECM, mainly through focal adhesions. Positive feedback between focal adhesions and integrins, the cytoskeleton, and the nucleus promote the expression and translocation of EMT and YAP/TAZ transcription factors to the nucleus.^87^ Other transcription factors that are relevant in cardiac EMT are: Tcf21,^88^ which is regulated by Wt1 and for which knock-out models showed downregulation of SNAIL and ZEB;^89^ Tbx18, which is important during heart developmental EMT;^90^ and serum response factor (SRF)/myocardin-related transcription factor tandem, which is essential for cell motility and contractility during developmental EMT.^91,92^

Interestingly, the epicardium becomes dormant after birth. However, cardiac injury later in adult life reactivates developmental gene programs that stimulate EMT in the epicardium.^93^ In particular, the Wnt1/β-catenin pathway has been reported to be activated after acute MI and to trigger epicardial EMT that derives in fibroblasts formation.^94^ Formation of other cardiac cells such as smooth muscle cells and coronary vascular cells have been also reported^95^ (Fig. 2). Using MI models in adult mice, genetic knock-out of epicardial YAP/TAZ—which causes EMT inhibition—led to persistent inflammation, widespread fibrosis, heart failure and death.^33^ Therefore, it is widely assumed that EMT in the adult heart is needed for better outcomes after MI. However, the human adult epicardium responds poorly to EMT when compared to the more active fetal epicardium under the same EMT-activation stimuli. In this sense, human epicardial cells from fetal and adult hearts were collected and stimulated *in vitro* with TGF-β. Polymerase chain reaction array analysis showed that unstimulated fetal epicardial cells clustered together with TGF-β stimulated fetal cells, whereas unstimulated adult cells clustered far away from TGF-β stimulated adult cells.^96^ This suggests that fetal and adult epicardial cells are in different states of activation, where fetal epicardial cells are closer to a mesenchymal state and therefore likely much more prone to undergo a reparative EMT process. In any case, adult epicardial cells treated with TGF-β are in an intermediate state between non-stimulated adult epicardial cells and highly reactive TGF-β-stimulated fetal cells, indicating that EMT induction in adult epicardium is a possibility. The question remains why adult epicardial cells have partially lost this activation potential, and if it could be fully re-established to assist the repair of the heart after MI as well as improvement of cell-based therapies.

## IV. BIOENGINEERING STRATEGIES TO CONTROL EMT

Various approaches have been used to induce or block EMT processes both to study it at a cellular and mechanistic level, but also to mimic the processes that can lead to organ development or possibly regeneration. In this section, we describe the methods and platforms that have been employed to recapitulate or control the EMT process. These bioengineering strategies can be broken down into approaches that involve biological agents, incorporate biochemical components and biomaterials, or introduce physical features and factors to control the EMT process.

### A. Biological approaches

A common approach to control EMT in cells is to provide the growth factors or cytokines involved in inducing or blocking the process in an *in vitro* setting. Since EMT is a normal process for many cells during development, and protocols for differentiation of stem cells and progenitor cells into specific cell types are typically modeled off the developmental process, this is a widely used tool in stem cell differentiation.^64^ While the first study to culture primary epicardial cells from human adults demonstrated that they can spontaneously undergo EMT *in vitro,*^58^ enhanced control over the process and the possibility of generating desired cell fates can be achieved by supplementing with key factors in a soluble form and along precise temporal scales. Indeed, it is understood that growth factor pathways are responsible for the cellular transformations during EMT.

Witty *et al.* established the formation of epicardial cells from human pluripotent stem cells through activation of the BMP and Wnt signaling pathways.^97^ Subsequently, they demonstrated that the hESC-derived epicardial cells could then undergo EMT and give rise to cells with characteristics of smooth muscle cells and fibroblasts. The treatment regimen to induce EMT involved exposure to TGF-β1 and basic fibroblast growth factor (bFGF), with cells specifying toward a smooth muscle-like fate with TGF-β and a fibroblast-like fate with bFGF. This ability to control the EMT process could provide a source of cells for cardiac tissue engineering, as well as potential routes for therapy for heart failure, and indicates that controlling the EMT progress through the use of growth factors is an effective approach.

The most powerful known inducer of EMT is TGF-β. The TGF-β superfamily is involved in a signaling network that regulates EMT and EndMT,^28,98^ and it consists of a group of ligands that includes TGFβs, bone morphogenetic proteins (BMPs), activins, inhibins, and growth and differentiation factors.^78,99^ Isoforms that activate signaling in EMT and EndMT include TGF-β1, 2, and 3, and BMP2 through BMP7, although they are associated differently with various EMT processes in development and disease and in various tissues,^59^ including the heart.^78^ Ligands of the TGF-β superfamily bind to a heterotetrameric receptor complex.^98^ The TGF-β receptor complex transduces a signal through phosphorylation of SMAD transcription factors, which then bind to promoters of EMT and EndMT inducing genes in the nucleus, including the key transcription factor families SNAIL, TWIST, and ZEB.^52,59,98^ TGF-β also regulates EMT and EndMT though non-SMAD signaling pathways. Of note as it relates to bioengineering control, is that TGF-β mechanisms involve mechanosensing. TGF-β bound in the ECM as a latent complex can be activated in response to integrin binding and tensile force exerted by integrins.^99^

Other molecules involved in control of EMT through various overlapping signaling pathways include FGF, insulin-like growth factor (IGF), platelet-derived growth factor (PDGF), and hepatocyte growth factor (HGF).^93^

Another important factor that has also been shown to induce EMT is the molecule thymosin β4 factor (Tβ4). Tβ4 is a 43-amino-acid peptide expressed in the developing heart^100^ and is highly conserved in most species.^101^ It is a pleiotropic protein, with its main function being sequestration of G-actin monomers. Additionally, Tβ4 is an angiogenic factor, a chemoattractant, and a cardioprotective biomolecule. Tβ4 can be found everywhere in the nuclei and cytosol of tissue and circulating cells except red blood cells.^100–102^ Importantly, it was shown that Tβ4 plays an essential role in the key stages of cardiac vessel development (vasculogenesis, angiogenesis, and arteriogenesis), to promote survival of cardiomyocytes, and to stimulate migration of cardiomyocytes and endothelial cells.^102^ During development, Tβ4 is expressed by the myocardium and acts in paracrine signaling for epicardium-derived cells to migrate and differentiate into coronary vascular smooth muscle cells.^20^ In the heart of zebrafish, Tβ4 was shown to increase EMT in cardiac valve formation.^103^ When Tβ4 is overexpressed in colon carcinoma cells, EMT occurs with downregulation of E-cadherin by ZEB1-mediation transcriptional repression and an accumulation of β-catenin, mediated by integrin-linked kinase (ILK) and Akt.^104^ Indeed, Tβ4 prompts cell migration and invasion through positive regulation of the ILK/Akt/β-catenin/integrin signaling cascade in human colorectal cancer cells.^105^ Tβ4 has demonstrated to have an inducing effect on EMT in other cancerous cells as well, such as OSCC,^106^ melanoma,^107^ urothelial carcinoma,^108^ and hepatoblastoma metastasis.^109^

**FIG. 3.**
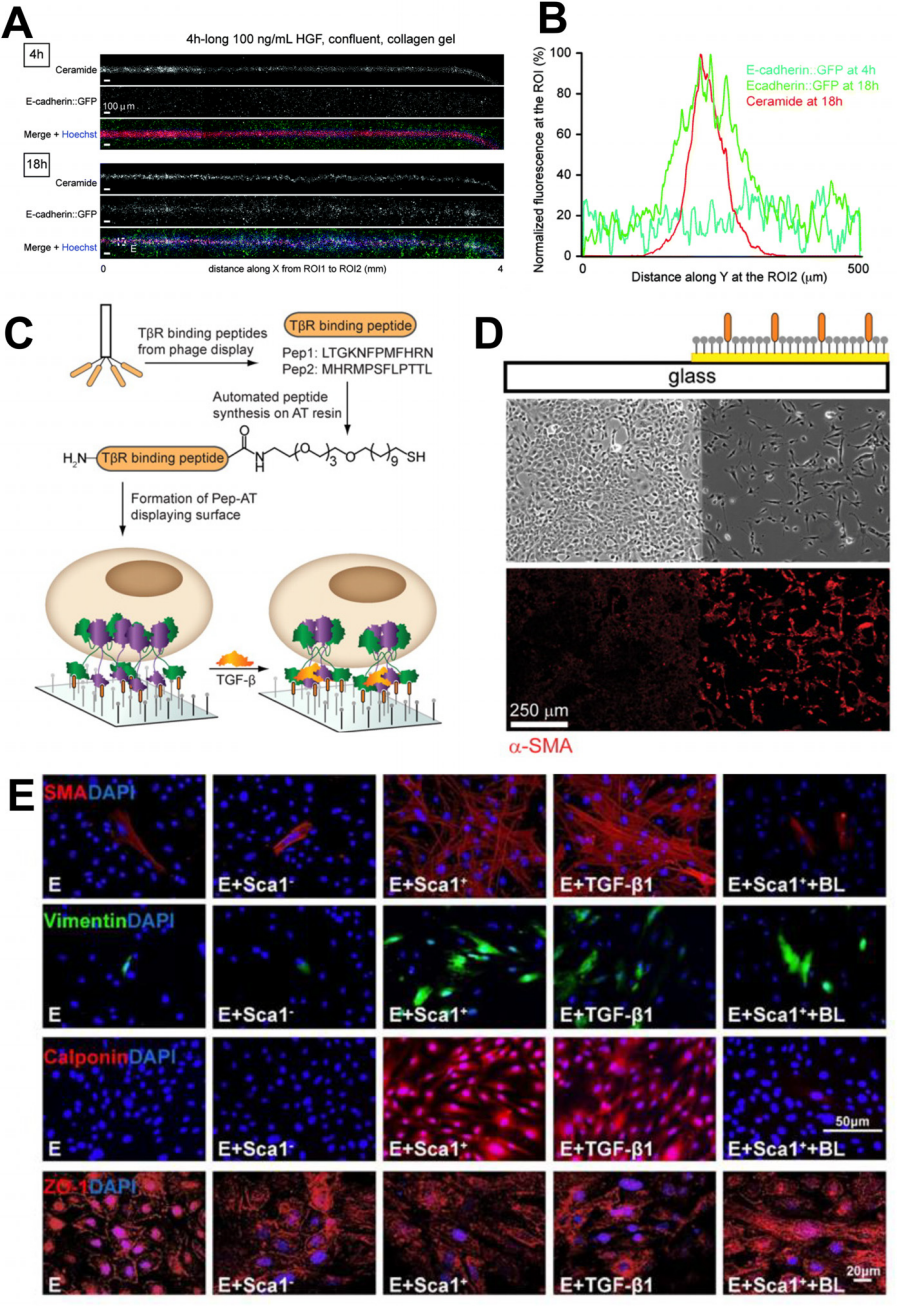
Biological approaches to control EMT. (a) Live-imaging of monolayer on collagen gel exposed to local HGF treatment (100 ng/ml) for 4 h. Cytoplasmic E-cadherin-green fluorescent protein (GFP) levels increase 18 h after treatment. (b) Fluorescence measurements indicated in line scan of samples in E at 4 and 18 h following treatment.^110^ Reproduced with permission from Benedetto *et al.*, Lab Chip **14**, 1336 (2014). Copyright 2014 Authors, licensed under a Creative Commons Attribution (CC BY) license. (c) Approach involving a self-assembled monolayer (SAM) composed of an alkanethiol (AT) displaying a TβR-binding peptide to establish preorganized TGF-β signaling complex. (d) NMuMG cells cultured on the surface patterned with peptide-substituted SAM demonstrate spatial control of cell fate. Cells were stained for α-SMA and indicate cells on the functionalized surface underwent EMT.^111^ Reproduced with permission from Li *et al.*, Proc. Natl. Acad. Sci. **108**, 11745 (2011). Copyright 2011 Authors, licensed under a Creative Commons Attribution (CC BY) license. (e) Immunofluorescent images of epicardial-derived cells (e) co-cultured with Sca-1^−^ cells (E + Sca1^−^), Sca-1^+^ cells (E + Sca1^+^), TGF-β1 (E + TGF-β1) or Sca-1^+^ cells with TGF-β1 blocking antibody (E + Sca1^+^ + BL) under hypoxia for 72 h. Staining for mesenchymal markers [smooth muscle actin (SMA), vimentin, calponin], and epithelial tight junction protein (ZO-1), and nuclei (DAPI) was performed.^56^ Reproduced with permission from Li *et al.*, Theranostics **8**, 1766 (2018). Copyright 2018 Authors, licensed under a Creative Commons Attribution (CC BY) license.

Many studies investigating the influence of growth factors often introduce them into a system globally in soluble form. However, the precise location and gradient over time of these factors are critically important. In fact, *in vivo*, these factors are regulated in a precise spatiotemporal manner, such that only certain cells undergo EMT. This approach of achieving spatiotemporal control of growth factor presentation was shown in an *in vitro* approach using a microfluidic chip with a hydrodynamic flow-focusing design to create localized morphogen and growth factor concentrations.^110^ Local treatment of HGF and TGF-β1 induced EMT in Madin-Darby Canine Kidney II cells (MDCK II cells), observed by the accumulation in cytoplasmic E-cadherin (or reorganization of E-cadherin—to cytoplasm), reduction in ZO-1, and/or lack of acetylated-α-tubulin in cells in a targeted area [Figs. 3(a) and 3(b)].^110^ Similarly, surface patterning through the arrangement of TGF-β receptor (TβR)-binding peptides could control TGF-β signaling and cell fate. This was demonstrated by the fact that the EMT processes of various cell types were enhanced after TGF-β pathway activation in response to interaction with peptide ligands that were conjugated as self-assembled monolayers where the cells were cultured^111^ [Figs. 3(c) and 3(d)].

Another biological approach to control EMT processes is the use of non-coding RNA.^112^ MicroRNAs (miRNAs) are small non-coding RNAs that bind messenger RNA and achieve posttranscriptional regulation of cellular processes. Numerous miRNAs have been found to regulate the expression of EMT transcription factors described in Sec. III such as SNAIL, ZEB, and TWIST.^112,113^ For example, the miR-200 family is well-known for their involvement in EMT and has been investigated thoroughly.^112,114^ These miRNAs play a significant role in regulation of ZEB transcription factors and have been shown to prevent EMT and maintain the epithelial phenotype.^115–118^ Additional examples relevant to the heart include miR-21 and miR-31, which have been shown to modulate cardiac fibrogenic EMT.^119,120^ Moreover, let-7, one of the earliest discovered miRNAs, is associated with cardiovascular diseases and the inhibition of let-7 through antimiR administration following MI demonstrated an increase in EMT and epicardial cells in the border zone, and improved cardiac function due to positive remodeling.^121^

Exosomes, which are multivesicular bodies that can transport bioactive molecules such as proteins and miRNAs, have also been investigated in terms of how they can influence EMT processes. Injury, such as an MI, can cause the release of exosomes, enabling paracrine signals to be sent between cells. In this sense, it has been demonstrated that the pericardial fluid (PF) from MI patients induced epicardial EMT, which may have been a result of the glycoprotein clusterin, which was present in PF-exosomes from MI patients.^122^ Clusterin has been shown to be a mediator for TGF-β-induced EMT^123,124^ and treatment with clusterin in murine MI models resulted in improved myocardial function.^122^ However, clusterin has also been shown to be associated with negative left ventricular remodeling after MI,^125^ and its full potential remains unclear.

Interestingly, stem cell delivery can also be used to enhance EMT. Since bone marrow reconstitution with young mouse stem cell antigen 1^+^ (Sca-1+) bone marrow cells was found to improve cardiac regeneration after MI,^126^ further work was conducted to investigate whether this impact was related to EMT processes.^56^ *In vitro,* bone marrow Sca-1^+^ or Sca-1^−^ cells were co-cultured with epicardial-derived cells and *in vivo*, Sca-1^+^ or Sca-1^−^ cells from young mice were used to reconstitute aged mice. Results demonstrated that Sca-1+ cells promoted the EMT process of epicardial cells, that more young bone marrow Sca-1+ cells homed to the epicardium and resulted in increased host epicardium-derived cell EMT, and that TGF-β1 was a key modulator of the EMT reactivation [Fig. 3(e)]. This presents an interesting approach to reactivating the program in epicardial cells that may lead to repair.

### B. Biomaterial and biochemical approaches

Biomimetic biomaterials have been widely used in tissue engineering for replication of diverse types of both normal and diseased tissues. Thus, by engineering materials that promote native developmental biology cues and signaling, the degree and duration of the EMT event could be controlled. Similar to the way interactions between material surfaces and stem cells have been shown to control stem cell fate,^127^ approaches involving material surface chemistry can be used for EMT since the process involves loss of cell adhesion as cells becoming more migratory. For example, using a heterogeneous prostate cancer cell line, OPCT-1, which typically contains epithelial and mesenchymal cell subpopulations, enrichment of the epithelial phenotype was enabled through use of an amino-functionalized material (SiH-3AP).^128^ Furthermore, differences in adhesion of the subpopulations of the OPCT-1 cells to a fluoroalkylsilica culture surface indicated that the status of EMT or cell differentiation governs the adhesive capabilities of these cells.^129^

**FIG. 4.**
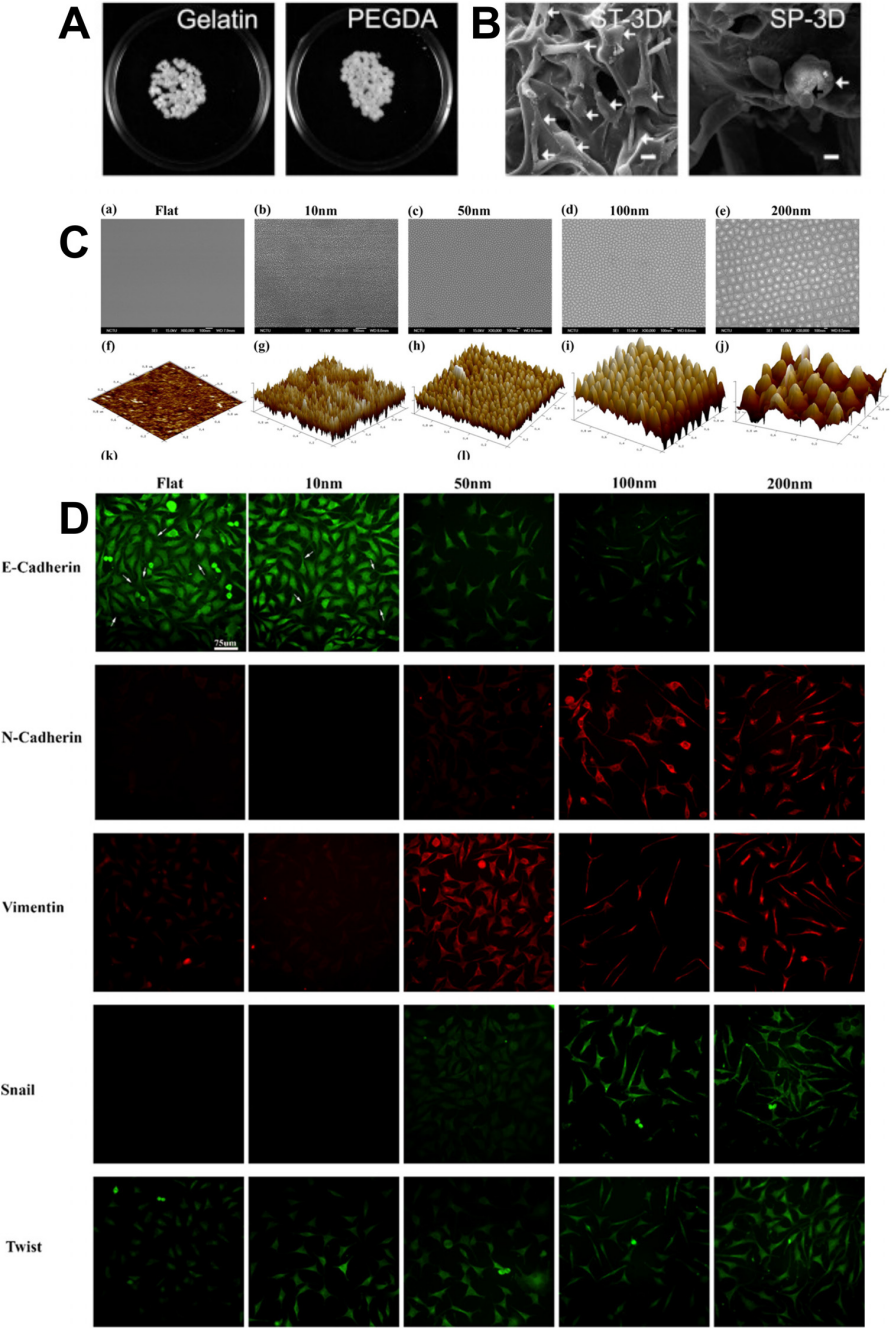
Biomaterial and physical approaches to control EMT. (a) and (b) Gelatin and poly(ethylene glycol) diacrylate (PEGDA) microscaffolds. Photographs of scaffolds (a) and SEM images (b) of HepaRG cultured in stretched 3D systems (ST-3D) and spheroid 3D systems (SP-3D). White arrows indicate HepaRG cells and black arrows indicate bile canaliculus between cells in spheroid (scale bar 20 *μ*m).^130^ Reprinted with permission from Wang *et al.*, Biomaterials **91**, 11 (2016). Copyright 2016 Elsevier. (c) Tantalum oxide nanodots ranging from 10 to 200 nm in diameter engineered in highly ordered pattern on Si substrates shown in SEM images (a)–(e) (scale bar 100 nm) and the corresponding AFM images (f)–(j). (d) Expression of EMT genes and transcription factors is demonstrated in fluorescent immunostaining of MDA-MB-231 cells on nanopatterned surfaces after 7 days (scale bar 75 *μ*m).^148^ Reprinted with permission from Dhawan *et al.*, ACS Appl. Mater. Interfaces **10**, 11474 (2018). Copyright 2018 American Chemical Society.

Another biomaterial approach involved investigating how EMT of hepatic cells was impacted by 3D culture systems compared to 2D culture systems with the difference relating to the composition of the scaffolds. Biomaterial-engineered EMT was achieved by culturing HepaRG in stretched 3D systems (ST-3D) using adherent scaffolds, spheroid 3D systems (SP-3D) using non-adherent scaffolds, and also compared to traditional 2D systems.^130^ Constrained EMT occurred in the spheroid 3D system, which corresponded to improved hepatic functions, and histone deacetylases was found to be a key factor in the EMT status^130^ [Figs. 4(a) and 4(b)].

The effect of material chemical compositions on the EMT process in cells has also been investigated. For example, reduced graphene oxide was shown to trigger EMT activation, having implications in terms of toxicity leading to pulmonary fibrosis,^131^ and the incorporation of silicate-based bioceramic particles into a nanofibrous composite scaffold for wound healing demonstrated evidence of activation of the EMT and EndMT signal pathways, and resulted in improved healing.^132^ Understanding the effect of specific material and chemical properties on EMT is important for understanding biological phenomena such as disease progression, but also can be highly useful for the design of engineered systems where control of EMT is desired.

Biomaterial scaffolds comprised of biologically derived ECM represent a promising approach in tissue engineering due to their chemically and mechanically relevant composition and structure. Studies that used biologically derived ECM as scaffolds have indicated that they can impact the EMT process. Decellularized human breast cancer scaffolds promoted EMT in the seeded MCF-7 cells, revealing that the ECM properties play a role in this process.^133^ Similarly, human liver cancer HepG2 cells grown on decellularized scaffolds of cirrhotic ECM showed an upregulation of EMT related genes and TGF-β signaling as compared to healthy ECM.^134^ In these cases, engineered scaffolds generated from diseased tissue possessed properties that induce EMT. Elements of the native ECM such as the physical structure (e.g., topographical or mechanical properties) and/or its biological and chemical cues could be important for EMT regulation and further investigation into the independent impact of these properties for various tissue sources will continue to provide insight into additional tools to control EMT for bioengineering approaches.

Hyaluronic acid (HA), an ECM component and a commonly used biomaterial, has been shown to play an important role in EMT. HA was shown to take part in the induction of EMT in lung and breast cancer cells through TGF-β1 or epidermal growth factor treatment.^135^ A study on zebrafish demonstrated that HA and hyaluronan-mediated motility receptor^136^ expression increased following cardiac injury and that HA could function with PDGF to promote epicardial EMT.^136^ Cardiac regeneration, which typically results from epicardial cells undergoing EMT and migrating into the site of injury, was blocked with chemical suppression of HA production and knockdown of Hmmr. In other work, TGF-β2 activation was linked to HA production and led to induction of EMT.^137^ Additionally, scaffolds composed of chitosan-hyaluronic acid showed increased expression of EMT-related genes compared to the control scaffolds.^138^ Indeed, HA signals may be a useful tool in altering EMT.

The use of instructive biomaterials that modify cellular adhesion may be a promising direction going forward for control of EMT. Transmembrane adhesion proteins are important for cell adhesion, with integrin involved in cell adhesion to the ECM, and cadherin involved in cell–cell adhesion. Designing biomaterials that incorporate or interact with these cell adhesion elements could then modulate cell behavior. For example, E-cadherin mimetic peptides immobilized on a gold surface demonstrated enhanced cell adhesion to the material surface and weakened cell–cell contacts.^139^ Similarly, material modification to achieve control over integrin function has also been explored.^140^ In one such study, cell-adhesive gold nanodots functionalized with arginine-glycine-aspartate peptides were used to pattern a surface, and their placement affected integrin clustering which impacts cell attachment and spreading.^141^ In addition, presentation or activation of growth factors via patterned biomaterials is another elegant approach to achieve control over the EMT process. An example of this is the work mentioned in Sec. IV A that demonstrated a patterned surface of peptide ligands able to bind to TGF-β receptors, activating TGF-β signaling and inducing EMT.^111^ Modifications of biomaterials with additional biological elements can achieve precise control over the microenvironment.

In terms of the *in vivo* microenvironments contribution to inducing EMT, not many studies have investigated this directly and independently. However, Bao *et al.* developed a cell differentiation protocol to achieve long-term self-renewing human epicardial cells and demonstrated that EMT and invasion of the myocardium took place *in vivo*. This was observed using cardiac-fibroblast-derived extracellular matrix patches seeded with hPSC-derived epicardial cells on the surface of mouse hearts.^142^

Finally, control over biochemical conditions such as oxygen concentrations has also been shown to impact EMT processes. EMT was promoted in mouse Tbx18-positive epicardial cells which differentiated into coronary vascular smooth muscle cells as a result of hypoxia, through hypoxia inducible factor-1alpha (HIF-1alpha)-mediated effects on Snail.^143^ The authors also showed that hypoxia led to premature differentiation of epicardial cells to coronary vascular smooth muscle cells and induced Snail expression *in vivo.* Likewise, other work with mouse cells demonstrated that hypoxia stimulated EMT and differentiation of epicardial cells into vascular smooth muscle cells in a manner involving the TGF-β pathway.^144^

### C. Physical approaches

While cell fate can be controlled by bioactive and chemical components, it is known that the physical properties of the materials can control their fate as well. Factors related to the ECM in which the cells live such as topology, alignment, and mechanical properties can impact cell behavior.^145^ Indeed, the niche environment is a well understood concept for stem cell differentiation,^146^ and this concept is relevant to the process of EMT as well.

The 3D microenvironment plays a significant role in cell processes and various physical properties, such as alignment and topography, are involved. It has been found that alignment in electrospun poly(ε-caprolactone) fibrous scaffolds altered the morphology and gene expression of breast cancer cells, indicating that these physical cues may induce EMT.^147^ Another physical approach to control EMT involved modifying the topography of the microenvironment. Again using breast cancer cells, engineered systems with tantalum oxide nanodots ranging from 10 to 200 nm showed induction of EMT in the 100 and 200 nm nanochips, as demonstrated by cell morphology and expression of EMT related proteins, transcription factors and gene expression^148^ [Figs. 4(c) and 4(d)].

Dimensionality and mechanical stiffness of the microenvironment may also impact EMT. CD44 is a membrane receptor for major ECM component HA, and its spliced isoforms control cell behaviors such as survival, growth and motility.^149^ As a result of culture within 3D hydrogels, gastric cancer cells exhibited variations in CD44 isoform expression, which was dependent on hydrogel matrix mechanical properties and corresponded with an upregulation in genes involved in EMT.^149^ However, a study that investigated the impact of gels composed of collagen and methacryaled HA (Coll-MeHA) with controlled mechanical properties demonstrated that there was enhanced EMT on the Coll-MeHA gels but that gel stiffness did not directly affect EMT.^150^ Furthermore, relating back to growth factor control over EMT, stiffness can also impact their effects. Work on the relationship between stiffness and EMT found that stiff fibronectin substrates induced EMT in alveolar epithelial cells, driven by increased cell contraction and integrin-mediated TGF-β activation.^151^ This can be related to the increased stiffness of fibrotic tissue for which EMT is a hallmark response. Substrate stiffness was found to have an effect on TGF-β1-induced cell function, with cells undergoing apoptosis or EMT depending on matrix rigidity.^152^ Epithelial cells treated with TGF-β1 responded based on matrix rigidity and a P13K/Akt-mediated switch was demonstrated. In addition, since latent TGF-β1 can be released from the ECM via tension,^153^ and in fact, mechanical pre-loading of the ECM can affect this latent TGF-β1 activation,^154^ there could be further implications in terms of TGF-β1 bioavailability through stiffness control.

## V. CONTROL OF EMT FOR CARDIAC TISSUE ENGINEERING APPROACHES

Modulation of EMT represents a highly valuable target in cardiac tissue engineering, as EMT could be harnessed for generating mesenchymal cells (MCs)^155^ with reparative potential.^93^ Thus, although EMT type 2 and 3 are generally associated with several cardiovascular pathological processes such as cardiac fibrosis or heart valve disease in adult mammals,^57^ we cannot ignore the ability of EMT type 1 to generate MCs that contribute to coronary vessel formation^156^ or positive valve remodeling^157^ during organ development. Therefore, EMT has the potential to contribute to ECM remodeling in a truly biomimetic fashion,^158^ ultimately determining the fate of cardiac muscle physiology. The question now is how to control and tune EMT in order to redirect adult EMT toward a reparative end and to produce the specific cardiac cell types needed for heart tissue repair.

The production of smooth muscle cells and fibroblasts by epicardial cells that undergo EMT in the adult heart has been taken advantage of as an experimental approach to improve heart repair following MI. As previously reviewed in Sec. IV, EMT can be induced in epicardial cells by a variety of growth factors, each one offering different outcomes. For instance, TGF-β1 enhances differentiation into smooth muscle cells,^159^ assisting the formation of blood vessels; basic FGF leads to fibroblasts formation,^97^ a process that is mainly associated with ECM remodeling; and Tβ4 appears to have a more broad action, with reports of inducing differentiation into fibroblasts, smooth muscle, and endothelial cells.^57^ From a cardiac healing perspective, EMT's main contribution to cardiac repair is in promoting neovascularization, which has been demonstrated in zebrafish^160^ and mouse^161^ models. Based on this premise, TGF-β1 and Tβ4, as well as any other growth factors able to induce EMT and promote angiogenesis such as FGF or HGF,^162^ may be candidates for inducing EMT and improving cardiac repair after infarction as well as the integration of cardiac patches. Very interestingly, zebrafish work showed that FGF-mediated EMT induction can result in increased neovascularization,^160^ although growth factors from the FGF family are generally related to cardiac fibrosis.^163,164^ In this regard, FGF17b isoform is increased in regenerating myocardium, along with FGF receptors 2 and 4^160^ and it is associated with angiogenic effects. On the other hand, other FGF isoforms like FGF21 and FGF23, and receptor FGF1c, prompt coronary artery disease and cardiac fibrosis.^163,165^ This indicates that inducing EMT is a challenging pathway to control, where slight modifications mean the difference between improving cardiac repair and worsening heart attack outcomes.

**FIG. 5.**
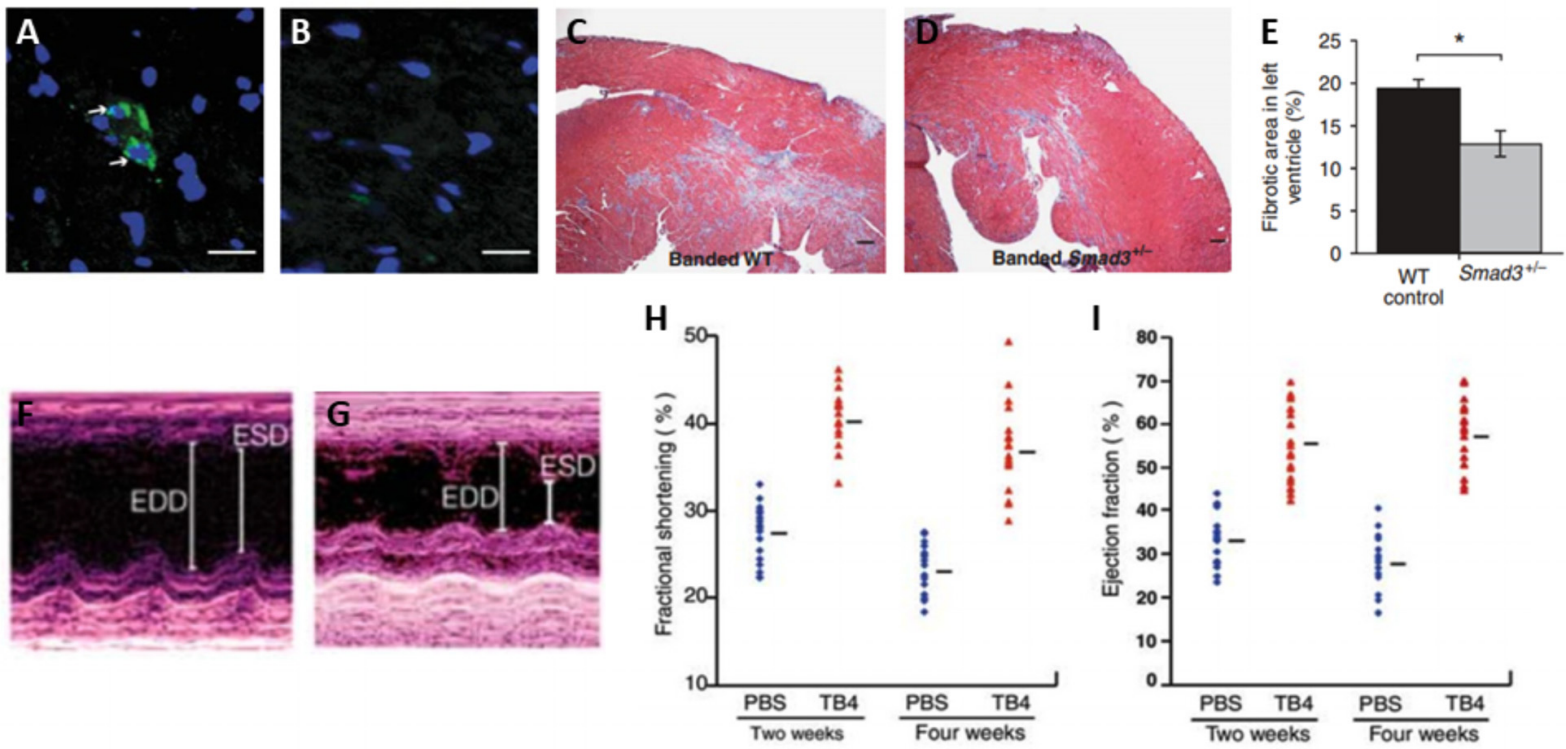
Different outcomes of EMT in the adult mouse hearts. (a) TGF-β1 expression was detected by immunofluorescence (green, arrows) in fibrotic heart sections of animals after MI induction. (b) TGF-β1 expression was not detected by immunofluorescence in non-fibrotic heart sections from animals with MI and sham surgery. Scale bars: 20 *μ*m. (c) Representative areas of Masson trichrome-stained banded hearts from a wild-type mouse. (d) Representative areas of Masson trichrome-stained banded hearts from a SMAD3± mouse (TGF-β1 pathway blocked). Scale bars: 200 *μ*m. (e) Morphometric analysis of cardiac fibrosis from wild-type I and SMAD3± animals indicating blockage of TGF-β1 pathway results in less fibrosis of the cardiac tissue.^166^ Adapted by permission from Zeisberg *et al.*, Nat. Med. **13**, 952 (2007). Copyright 2007 Springer Nature: Nature Publishing Group. (f) Representative echocardiographic M-mode image of the left ventricle after MI of a control animal (MI induction, no treatment). (g) Representative echocardiographic M-mode image of the left ventricle after MI of a Tβ4 treated animal. (h) Distribution of left ventricular fractional shortening at 2 and 4 weeks after MI in control animals [phosphate-buffered saline (PBS)] and Tβ4 treated animals. Bars indicate means. (i) Distribution of left ventricular ejection fraction at 2 and 4 weeks after MI in control animals (PBS) and Tβ4 treated animals. Bars indicate means.^100^ Adapted by permission from Bock-Marquette *et al.*, Nature **432**, 466 (2004). Copyright 2004 Springer Nature: Nature Publishing Group.

Another relevant example is the use of TGF-β1 for cardiac tissue repair. TGF-β1 role in heart development is well described, where it is the main mediator of the EMT process needed to generate cardiac cells to populate the developing heart,^57^ but in aged mammals TGF-β1 mediated endothelial to mesenchymal transition is responsible of negative vein graft remodeling,^28^ cardiac fibrosis^166,167^ [Figs. 5(a)–5(e)], as well as tumor development and progression via EMT.^168^ Consequently, FGF and TGF-β growth factors, despite being potent EMT enhancers, are not considered for experimental treatments for MI due to the serious side effects they may trigger.

A more recent research trend for inducing EMT for cardiac repair after MI is through the use of Tβ4. So far, Tβ4 proved to be efficient in small animal models by enhancing myocyte survival and improving neovascularization in the infarcted tissue,^169^ as well as improving cardiac function after MI^100,170^ [Figs. 5(g)–5(i)]. Tβ4 has also been reported to trigger epicardium-derived progenitor cell differentiation,^171^ that could help in restoring normal heart cell composition and function. Unfortunately, Tβ4 administration did not result in any cardioprotective effect in pig models of ischemia/reperfusion injury.^172^ Although in both small and large animal models intravenous Tβ4 administration was used, mice received the growth factor for 7 days prior to the induction of MI. On the other hand, pigs only received 30 min intravenous Tβ4 infusion before MI, and another 30 minutes Tβ4 infusion 6 h after surgery. Differences in dosage (i.e., lower in pigs) may be the reason because of which Tβ4 treatment failed in large animal models. Special attention must be put on the escalation toward more relevant animal models that resemble human physiology, as different dosage protocols (total amount of growth factor administered, time of administration and route of administration) determine the success or failure of the treatment. Altogether, this evidence highlights the importance of better understanding EMT in the adult heart, since if modulated and fostered properly, it holds the potential to repopulate the infarcted cardiac tissue and restore organ homeostasis. EMT could also improve the effectiveness of cardiac cell therapies and integration of cardiac patches by modifying the epicardial barrier between the implanted patch and the myocardium.

Recapitulation of *in vivo* environments *in vitro* has improved greatly with the advance of organ-on-a-chip technology.^45^ In terms of cardiac tissue engineering and regeneration, the ability to investigate the modulation of processes like EMT in an *in vitro* system that can more adequately convey the biological, chemical and physical properties involved *in vivo*, would be highly beneficial for elucidating favorable methods of EMT control, thus leading to potential regenerative therapies. The development of a platform that enables direct and independent modulation of variables to control EMT in the relevant cell types constructed to recapitulate more mature tissue could provide important information for tissue engineering approaches going forward.

## VI. NOVEL TOOLS TO CHARACTERIZE EMT

Commonly used approaches to indicate progression through EMT have involved investigating cell morphology, cell migration, and the presence of epithelial and mesenchymal markers at the molecular signaling and/or transcription levels. Common markers of the epithelial state include E-cadherin and ZO-1 at the cell surface, while mesenchymal markers include SNAIL, SLUG, Vimentin and α-SMA. Furthermore, the quantitative analysis of the up- and downregulated transcription factors and expressed proteins previously discussed are used to indicate progression through EMT.

However, recently, to more accurately characterize this cellular process, markers associated with EMT can be used to generate specific EMT signatures. This has been completed for numerous cancerous cell lines and tissue.^173–175^ In some cases, the ability to assess progression through EMT can be used to help decide therapeutic options, for example, in the case of non-small cell lung carcinoma (NSCLC).^176^ EMT gene expression signatures were developed for NSCLC cell lines in order to assess tumor EMT status and its association with drug response. Using microarray platforms and high-throughput functional proteomic profiling, genes with mRNA expression levels either positively or negatively correlated with at least one of four EMT markers (i.e., E-cadherin, vimentin, N-cadherin and/or fibronectin 1) and bimodal distribution patterns were used to generate a 76-gene EMT signature indicating whether a NSCLC cell line had undergone EMT. Furthermore, differences in drug response between the epithelial and mesenchymal cancers were shown.

Similar to lung carcinoma,^177^ other specific sets of genes that define EMT and cellular states have been reported for other cancer disorders such as neck squamous cell carcinoma,^178^ endometrial cancer,^179^ ovarian cancer,^180^ and bladder cancer.^181^ All these studies and others led to the creation of the first gene resource database for exploring EMT-related human genes in cancer in 2015.^182^ The efficacy of EMT gene signatures has been proven to be precise and applicable in clinics, as demonstrated in a recent study showing that the prognosis of head and neck squamous cell carcinoma (HNSCC) can be predicted when the identification of cancer EMT gene signature is detected.^178^ Comparing NSCLC-EMT and HNSCC-EMT gene signatures, four top candidates arise: E-cadherin, vimentin, N-cadherin and fibronectin 1. Specifically, E-cadherin expression was reduced during EMT, whereas vimentin, N-cadherin and fibronectin 1 were upregulated. Although this EMT gene signature (including genes derived from and related to the aforementioned top four genes) has not been applied to other tumors, it appears that it could be conserved among different disorders, potentially including cardiac EMT processes.

Control of cellular behavior depends on interactions in complex regulatory networks, including gene regulatory networks (GRNs) and interaction networks in biomolecules.^183^ Building on methods to reconstruct GRNs, an ensemble-based network aggregation (ENA) approach was developed, which can incorporate networks obtained through different methods and datasets and improve accuracy.^184^ As a sample, this approach was applied to the NSCLC dataset associated with EMT to build a gene regulatory network and three major nodes were identified as hub genes, indicating potential drug targets.^184^ In terms of the heart, mass spectrometry and bioinformatics were integrated to generate a regulatory network to predict signal transduction nodes within the epicardium and myocardium, identifying the NF-κB node to be essential for cardiac EMT.^80^

Another approach that is gaining attention by the scientific community to better characterize and monitor EMT is the use of reporter lines that label epicardial and/or mesenchymal cells, ideally along with their progenies. Currently, several reporter cell lines that fluorescent label E-cadherin or vimentin (markers for epicardial and mesenchymal cells, respectively) have been developed and are currently available for purchase. These include human cell lines A549 vimentin red fluorescent protein (epithelial cell line), HCT116 vimentin RFP (colorectal cancer), BT-474 E-cadherin EmGFP (breast cancer), and MCF10A E-cadherin EmGFP (breast epithelial cells). These *in vitro* systems are a great tool for understanding the basic mechanisms underlaying EMT. However, they do not represent the complex *in vivo* microenvironment and tissue architecture, making them not the most accurate models for fully addressing EMT. Using a similar approach, *in vivo* reporter lines for tracking EMT generally target cells with specific epicardial markers. Using Cre/Lox systems it is possible to permanently label those cells and their progeny. One of the most commonly used epicardial markers is Wt1. Thus, Wt1^Cre^ based reporter lines have been successfully used in a number of studies for reporting what cells are derived from cardiac EMT, mainly identifying fibroblasts and smooth muscle cells.^38^ Importantly, Wt1 reporter line has also been employed in MI models to determine whether EMT is induced after MI as a defense mechanism. Today, there is evidence provided by *Wt1^CreERT^*^2/+^; *Rosa*26^*mTmG/+*^ reporter mouse model indicating that EMT induction after MI is scarce and mainly contributes to fibroblasts formation.^185^ More sophisticated reporter lines have been developed, as is the case of the mouse mammary tumor virus encoding the Polyoma Virus middle T antigen, Rosa26-RFP-GFP, and Fsp1-Cre triple transgenic mouse model.^186^ Using this approach, it is possible to assess the conversion of RFP epithelial cells to GFP mesenchymal cells under the control of the Fsp1 promoter, a gatekeeper of EMT initiation. Interestingly, using similar approaches but changing the promoter to other EMT regulatory genes (i.e., WT1, Tbx18, Tcf21, Gata5, Sema3d, Scx, or Sema3d^38,79^) would allow to further study how other regulatory pathways affect EMT and identifying new therapeutic markers.

Generating EMT signatures, uncovering novel pathways and hub genes involved in EMT processes, and optimizing reporter lines could very likely indicate areas where regulation of the EMT progress may be achieved, thus supporting bioengineering strategies to control EMT going forward. For cardiac tissue, analysis of EMT of epicardial tissue using these tools could provide useful information on the key pathways that can be targeted in therapeutic approaches to treat MI.

## VII. PERSPECTIVES AND FUTURE WORK

EMT is a widespread cellular process that occurs in development, disease and regeneration. Understanding the role and mechanism of EMT in each one of these processes provides important and distinct lessons for bioengineers. Having a thorough understanding of EMT in development provides information on pathways that can be mimicked or modified to achieve stem cell differentiation, cell reprogramming, and building tissue constructs. Gaining insight into how and why EMT occurs in the context of disease can elucidate ways to prevent or reverse those negative effects which can lead to disease treatment options. Finally, by investigating the role of EMT in regeneration, its potentially advantageous function can be harnessed in regenerative strategies to treat damaged tissues and organs and to generate engineered tissues. For regeneration as it relates to the heart, production of fibroblasts must be controlled, whereas the creation of other EMT derived cells such as smooth muscle cells or endothelial cells must be enhanced. Moreover, the interaction of the epicardial layer, EMT and immune system (especially heart resident macrophages) may be of utmost importance for optimizing and improving cardiac tissue repair.^41,187^ The common thread in each of these cases is that to achieve the desired outcomes, control over the EMT process must be achieved, and this relies on the use of bioengineering strategies.

**FIG. 6.**
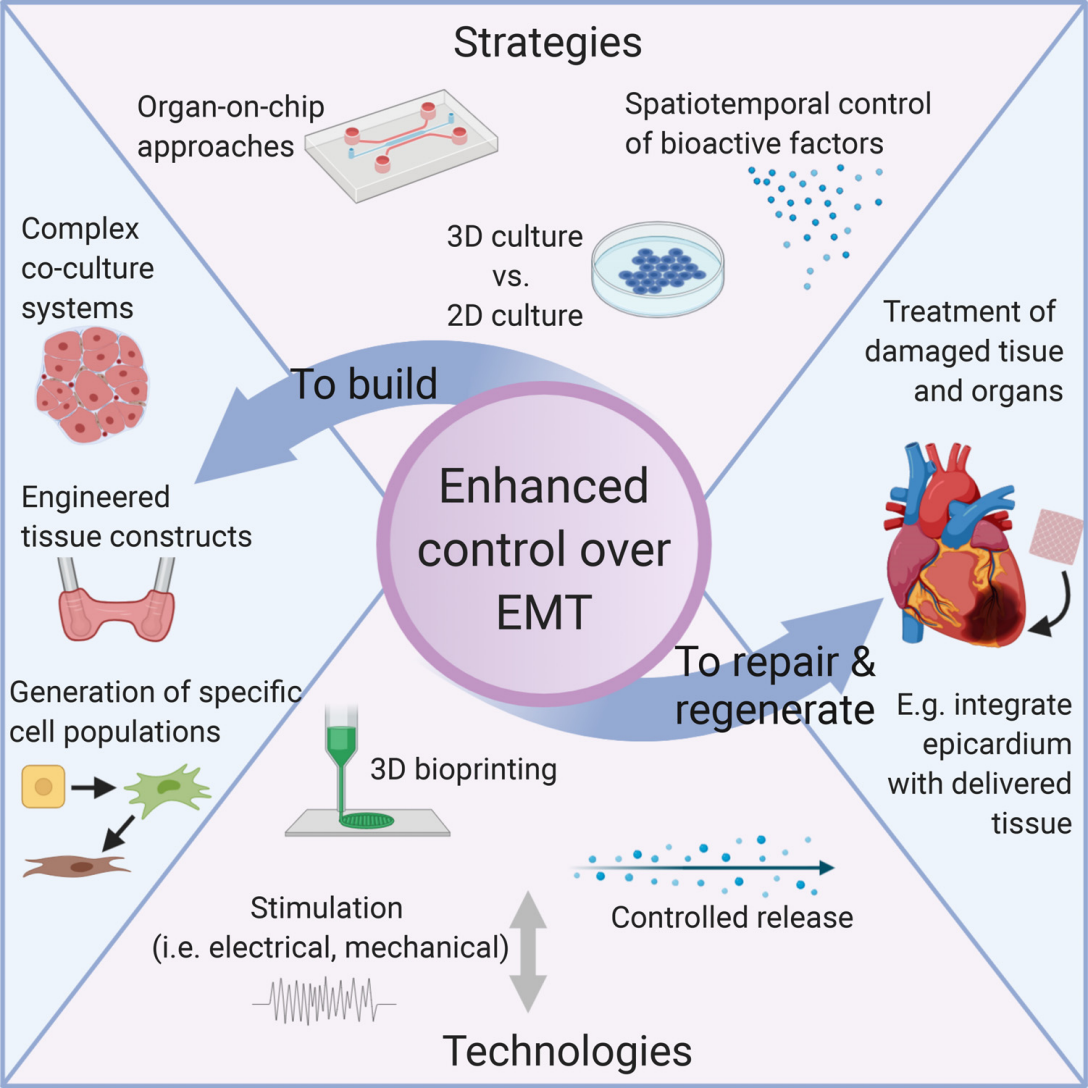
Strategies and technologies to achieve enhanced control over EMT in order to build cell and tissue constructs and to repair and regenerate damaged tissue. Figure created with BioRender.com.

The strategies to control EMT discussed in this review include biological, biomaterial and biochemical, and physical approaches (Fig. 6). While biological approaches to date have made use of bioactive factors and cellular components to induce EMT, enhanced spatiotemporal control over these factors could lead to better recapitulation of native microenvironments such that precise cell fates can be achieved, and more complex tissues can be generated. For this, controlled delivery methods can be utilized to present the cocktails of factors to suit the specific requirement at a relevant release rates and gradients. In terms of biomaterial and biochemical approaches to control EMT, future work should incorporate more organ-on-a-chip bioengineering strategies. It is well established that 3D tissue culture systems demonstrate more physiologically relevant properties and can provide useful information in assays compared to 2D tissue culture.^45^ Therefore, to ensure the basic biological investigations of EMT are demonstrating results that correspond to the native *in vivo* situations, these investigations should be carried out in 3D systems using biomaterials that mimic the 3D microenvironment. Furthermore, for the goal of building functional tissue engineered constructs, where EMT may be utilized to mimic developmental processes, it is imperative that the 3D microenvironment is incorporated to achieve dimensionally relevant structures. It is also important that in these organ-on-a-chip systems, appropriate physical properties such as mechanical and electrical stimulation are considered and integrated, to achieve more mature tissue. Bioengineering techniques such as surface functionalization, instructive biomaterials, and 3D printing can also assist in generating precisely pattered surfaces and organized cellular constructs to support these objectives.

It is possible that with enhanced control over EMT, the process will be increasingly relied upon and built into protocols to generate specific cell populations. For example, recently, cardiac fibroblasts were established from epicardial cells via EMT and used in tri-cellular culture to generate cardiac microtissues.^188^ With this strategy in mind, it may be possible that complex co-culture systems that resemble native tissue in terms of cell populations could be developed by relying on EMT *in vitro* to establish the various components of the tissue.

In the context of cardiac tissue engineering, there are opportunities going forward for enhanced control of EMT to improve the integration of tissues delivered to the damaged heart for regeneration. Cell injection as a regenerative strategy suffers from significant cell death and washout following delivery^189,190^ as well as limited functional integration.^191,192^ Biomaterial patches and engineered tissue patches which are delivered to the epicardial surface of the heart may circumvent the issue of the cell death and washout, however, their integration with the heart remains a challenge.^193^ With enhanced control over the EMT process, it may be possible that the epicardial cells present on the heart surface can be encouraged to transform and integrate with the replacement tissue. Furthermore, in either case of cell injection or cardiac patch delivery, if EMT can be sufficiently controlled, the epithelial cells present in the heart may be able to give rise to cardiac fibroblasts and coronary smooth muscle cells, which could lead to improved integration with replacement cells or tissue, as well as enhanced vascularization of the region.

## VIII. SUMMARY

The process of EMT wherein epithelial cells transform into a mesenchymal cell type is highly relevant to many biological processes involved in both development and disease. In this review, we first discussed the cellular processes and molecular mechanisms involved in EMT, as an understanding of the integrated networks of regulation is necessary in order to achieve control over the process. We reviewed the bioengineering approaches that have been undertaken to control EMT and described methods that employ biological agents and alter the material, biochemical, and physical properties of cellular microenvironments to induce or otherwise alter the EMT process. Since EMT occurs in diseased tissue, achieving control over the process is sought after for the development of novel therapies. One important example of EMT in disease is the advance of cardiac fibrosis following severe injury in the heart, and thus, approaches that have been used to control EMT specific to cardiac tissue engineering were also described. It is clear that going forward, advanced bioengineering strategies to control EMT will be highly useful in both tissue engineering and regenerative medicine.

## Data Availability

Data sharing is not applicable to this article as no new data were created or analyzed in this study.
